# Lignin–Hydroxypropyl-β-Cyclodextrin Matrix for Chlorzoxazone Loading and Release: Physicochemical, Antioxidant, and Antimicrobial Characterization

**DOI:** 10.3390/polym18192429

**Published:** 2026-10-05

**Authors:** Andreea Creteanu, Narcis Anghel, Cornelia Vasile, Gabriela Lisa, Claudiu N. Lungu, Graţiela Popa

**Affiliations:** 1 Department of Pharmaceutical Technology, Faculty of Pharmacy, “Grigore T. Popa” University of Medicine and Pharmacy, 16 University Street, 700115 Iasi, Romania; eliza.popa@umfiasi.ro; 2 Laboratory 5 Natural Polymers, Bioactive and Biocompatible Materials Petru Poni Institute of Macromolecular Chemistry, 41A Gr. Ghica Voda Alley, 700487 Iasi, Romania; anghel.narcis@icmpp.ro; 3 Department of the Physical Chemistry of Polymers “Petru Poni” Institute of Macromolecular Chemistry, Romanian Academy, 41A Gr. Ghica Voda Alley, 700487 Iasi, Romania; cvasile@icmpp.ro; 4 Department of Chemical Engineering, Faculty of Chemical Engineering and Environmental Protection, “Gheorghe Asachi” Technical University, 73 Prof. Dimitrie Mangeron, Street, 700050 Iasi, Romania; gapreot@ch.tuiasi.ro; 5 Department of Functional and Morphological Science, Faculty of Medicine and Pharmacy, Dunarea De Jos University, 800008 Galati, Romania

**Keywords:** chlorzoxazone, inclusion complexes, lignin, cyclodextrin, drug delivery matrix, antioxidant, antimicrobial activity

## Abstract

Chlorzoxazone (CLZ) is a poorly water-soluble drug whose formulation may benefit from carrier systems capable of drug incorporation and controlled release. This study investigated an epichlorohydrin (ECH)-mediated lignin–hydroxypropyl-β-cyclodextrin (LIG/HP-β-CD) matrix for CLZ loading and release and evaluated its physicochemical, antioxidant, and antimicrobial characteristics. The resulting material was characterized by Fourier-transform infrared spectroscopy (FTIR) and thermogravimetric analysis (TG/DTG), while CLZ release was evaluated using kinetic models. The experimentally determined CLZ loading was 41.8 mg/g, corresponding to a loading efficiency of 20.9% relative to the initially added drug. After 180 min, the cumulative amount released reached 29.81 mg/g, corresponding to 71.32% of the loaded CLZ. The release profile was adequately described by the Weibull model (R^2^ = 0.99394), which provided a useful empirical description of the observed release behavior. FTIR analysis showed changes in the relative contributions of oxygen-containing functional-group regions after modification, while TG/DTG demonstrated an altered thermal profile of the CLZ-loaded material compared with the individual components; these findings were interpreted as supportive rather than definitive evidence of drug–matrix interactions. DPPH radical-scavenging activity was 41.82 ± 1.07% for LIG and 50.32 ± 1.05% for LCD–CLZ; however, because the samples were tested at different concentrations, these values were not interpreted as demonstrating an intrinsic enhancement of antioxidant potency. Under the agar-diffusion conditions, LCD–CLZ produced measurable inhibition against *Staphylococcus aureus*, *Escherichia coli*, and *Candida albicans*, whereas no effective inhibition was observed against *Pseudomonas aeruginosa*. Overall, the results support further investigation of the LIG/HP-β-CD matrix as a potential platform for CLZ loading and release, while additional structural, biocompatibility, and formulation-specific studies are required before pharmaceutical application can be established.

## 1. Introduction

**Figure 1:**
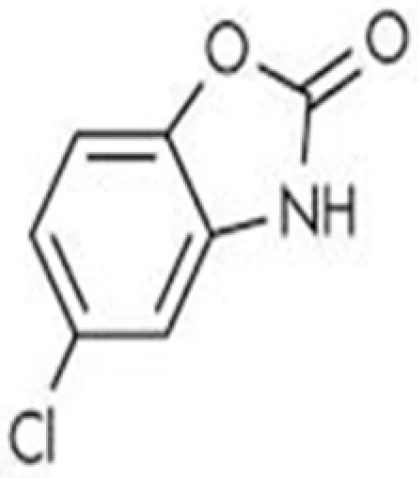
Chemical structure of chlorzoxazone (CLZ; 5-chloro-2,3-dihydro-1,3-benzoxazol-2-one).

Chlorzoxazone (CLZ; 5-chloro-2,3-dihydro-1,3-benzoxazol-2-one) is a centrally acting skeletal muscle relaxant used in the management of painful musculoskeletal conditions associated with muscle spasm [1,2,3]. CLZ is rapidly absorbed and extensively metabolized in the liver, predominantly via CYP2E1 [4,5]. From a formulation perspective, an important limitation is its poor aqueous solubility, combined with relatively high membrane permeability, characteristics consistent with a Biopharmaceutics Classification System (BCS) class II drug [6,7,8]. For poorly water-soluble compounds such as CLZ, formulation and dissolution characteristics may influence drug availability, supporting the investigation of carrier systems capable of improving drug incorporation and controlling its release [9] (Figure 1).

Cyclodextrins (CDs) are widely investigated as carriers for poorly water-soluble compounds because their hydrophobic internal cavity can accommodate lipophilic molecular regions, whereas their hydrophilic external surface promotes compatibility with aqueous environments [10,11,12,13,14,15]. β-cyclodextrin and its derivatives have therefore been used to form inclusion systems with several poorly soluble drugs [16]. Hydroxypropyl-β-cyclodextrin (HP-β-CD), used in the present study, combines the host–guest properties of the cyclodextrin cavity with improved aqueous compatibility. Incorporating cyclodextrin functionality into a polymeric matrix may also provide a means to modulate drug retention and release.

Lignin (LIG) is an abundant aromatic biopolymer containing phenolic and aliphatic hydroxyl groups and other reactive functionalities. Its structural versatility, together with reported antioxidant and antimicrobial properties, has stimulated interest in lignin-based pharmaceutical and biomedical materials [10,17,18,19,20,21,22,23,24,25]. Lignin has been investigated as a component of drug-delivery systems, including particulate and polymeric matrices capable of drug incorporation and controlled release. Combining lignin with cyclodextrin functionalities is particularly attractive because it may integrate the structural and functional properties of lignin with the molecular-recognition characteristics of cyclodextrins.

Epichlorohydrin (ECH) can be used as a coupling or crosslinking reagent in lignin/cyclodextrin systems. Previous work has demonstrated the potential of lignin–cyclodextrin matrices for drug delivery, including systems investigated for ketoconazole and piroxicam, in which drug retention and controlled-release behavior were reported [26]. Other approaches have similarly exploited the grafting of β-CD onto lignin-based materials [27]. These studies establish the feasibility of lignin/cyclodextrin platforms but also indicate that their performance depends on the chemical composition of the matrix, the incorporated drug, and drug–matrix interactions.

Although cyclodextrin-based formulations and lignin/cyclodextrin matrices have previously been investigated as drug-delivery platforms, the behavior of CLZ within an ECH-mediated lignin/HP-β-CD matrix remains insufficiently characterized. The specific contribution of the present study therefore lies in evaluating this combined matrix for CLZ loading and release, together with its physicochemical and functional characteristics. The resulting material was characterized by FTIR, thermogravimetric analysis, CLZ loading and in vitro release were evaluated, and the release profile was analyzed using kinetic models. Antioxidant and antimicrobial activities were additionally investigated as functional characteristics of the resulting LCD–CLZ system. The study was designed as a preliminary physicochemical and functional characterization rather than as an evaluation of a final pharmaceutical dosage form.

## 2. Materials and Methods

### 2.1. Materials

Chlorzoxazone (CLZ; 5-chloro-2,3-dihydro-1,3-benzoxazol-2-one) was obtained from Orchid Chemicals Ltd. (Chennai, India). Alkali lignin (LIG), hydroxypropyl-β-cyclodextrin (HP-β-CD), epichlorohydrin (ECH), and 2,2′-diphenyl-1-picrylhydrazyl (DPPH) were obtained from Sigma-Aldrich, St. Louis, MO, USA. Tetraethylammonium bromide (TEAB), sodium hydroxide (NaOH), hydrochloric acid (HCl), ethanol, and the other reagents and solvents used in the synthesis and analytical procedures were of analytical grade. All reagents and solvents were used as received without further purification unless otherwise specified.

### 2.2. Synthesis of Lignin–HP-β-CD Conjugate (LCD)

Synthesis of the lignin–hydroxypropyl β-cyclodextrin conjugate (LCD) follows three steps previously described [26,28], but the conditions have been modified to improve the quality of the final products.

#### 2.2.1. Step 1, In Situ: Epoxidation of the Alkaline Lignin

Alkaline lignin (3.00 g, technical grade, dried at 50 °C under vacuum overnight) was dispersed in 60 mL of aqueous NaOH (12 wt%) in a 250 mL three-neck round-bottom flask equipped with a mechanical stirrer, thermometer, reflux condenser, and dropping funnel. Tetraethylammonium bromide (TEAB; 60 mg, approximately 2 wt% relative to lignin) was added as a phase-transfer catalyst. The mixture was heated to 40–50 °C and stirred for 30 min until a homogeneous brown suspension was obtained.

The reaction mixture was subsequently cooled in an ice-water bath to 5–10 °C. Epichlorohydrin (1.20 mL, corresponding to approximately 1.2 equivalents per nominal lignin hydroxyl group) was added dropwise over 30–40 min under vigorous stirring while maintaining the internal temperature below 15 °C. After completion of the addition, the ice bath was removed and the mixture was allowed to warm to 30–35 °C. The suspension was stirred at 30–35 °C for 1.5–2.0 h under strongly alkaline conditions (pH ≥ 12). The resulting epoxide-functionalized lignin intermediate was used directly in the subsequent coupling reaction without isolation.

#### 2.2.2. Step 2: Coupling with HP-β-CD

Following the epoxidation stage, the reaction mixture was adjusted to approximately 35–40 °C, and 30–40 mL of deionized water was added to reduce the NaOH concentration from 12 wt% to approximately 8–10 wt% while maintaining strongly alkaline conditions. Separately, HP-β-CD (2.50 g) was dissolved in 25–30 mL of deionized water at 30–40 °C under magnetic stirring until a clear solution was obtained.

The HP-β-CD solution was added dropwise to the lignin-containing reaction mixture over 10–15 min at 35–40 °C under vigorous mechanical stirring. The reaction temperature was subsequently increased to 50–55 °C and maintained for 4–6 h. The pH was maintained at approximately 12 during the reaction, with minor additions of concentrated NaOH solution when required.

#### 2.2.3. Work-Up and Purification

Upon completion of the coupling step, the reaction mixture was cooled to room temperature and cautiously neutralized with aqueous HCl (2 M) under stirring to pH 3.5–4.0. The solid LIG–HP-β-CD (LCD) material was recovered by vacuum filtration using a medium-porosity glass filter. The filter cake was washed repeatedly with distilled water, followed by ethanol, to remove soluble unreacted HP-β-CD, inorganic salts, residual reagents, and low-molecular-weight reaction products. The washed material was dried at 40–50 °C under reduced pressure overnight and stored in a desiccator at room temperature, protected from light, until further characterization. Although this purification procedure was designed to remove soluble unreacted components, complete removal of residual epichlorohydrin and quantitative incorporation of HP-β-CD were not independently determined in the present study.

### 2.3. Loading of Chlorzoxazone into LCD Matrix

A total of 1 g of LCD was stirred for 24 h with 50 mL of ethanol containing 200 mg CLZ. The solid was filtered and dried at 40 °C in a vacuum space. The amount of CLZ encapsulated in cyclodextrin was spectrophotometrically determined by using the absorbance of the solution before and after filtration at 280 nm, measured using a Jenway 6405 UV–Vis spectrophotometer (Jenway Ltd., Dunmow, UK).

### 2.4. Investigation Methods

#### 2.4.1. Fourier-Transform Infrared Spectroscopy (FTIR)

ATR-FTIR spectraBruker (Optik GmbH, Ettlingen, Germany) were recorded using a Bruker Vertex 70 FTIR spectrometer equipped with a ZnSe attenuated total reflectance (ATR) accessory at an incidence angle of 45°. Spectra were acquired over the 4000–400 cm^−1^ range at a spectral resolution of 2 cm^−1^, with 64 scans averaged for each spectrum.

For semi-quantitative comparison, integrated band-area ratios were calculated relative to the lignin aromatic region at 1500–1605 cm^−1^. The spectral regions evaluated were 1680–1740 cm^−1^ (carbonyl), 2850–2970 cm^−1^ (aliphatic C–H), 3200–3600 cm^−1^ (O–H), 950–1180 cm^−1^ (C–O/C–O–C), and 880–930 cm^−1^. These ratios were used only as semi-quantitative descriptors of relative spectral changes and not as absolute measurements of functional-group concentration or grafting efficiency. No peak fitting or spectral deconvolution was performed.

#### 2.4.2. Thermogravimetric Analysis

The analysis of thermal behavior under dynamic conditions of the individual components and of the complex was carried out in an inert atmosphere, namely nitrogen, at a flow rate of 20 mL/min, using a Discovery TGA 5500 (TA InstrumentsTA Instruments, New Castle, DE, USA) instrument. The mass of the samples subjected to analysis ranged between 3.2 and 6.3 mg. They were heated at a rate of 10 °C/min over the temperature interval of 17–600 °C. TG curves and their derivative (DTG) were recorded.

#### 2.4.3. Chlorzoxazone Release from LCD Inclusion Complex

In the designing of controlled-release systems, one of the key goals is to characterize how an active ingredient behaves/is loaded and released in its carrier matrix over time. Kinetic models offer a quantitative framework for describing this evolution: they make it possible to compare formulations using the same reference, identify the prevailing release mechanism, and guide formulation optimization when experimental variables change.

In pharmaceutical and nutraceutical studies, a set of semi-empirical or empirical expressions is frequently used, most notably being the Higuchi, Korsmeyer–Peppas, Makoid–Banakar, and Weibull models. While each model links released fraction (or amount) to time, they differ in what they “highlight”, ranging from strictly diffusion-dominated transport to situations where diffusion, swelling, erosion, and polymer relaxation act together.

The Higuchi model [29] is among the earliest, and still one of the most common, approaches used for diffusion-controlled release from solid matrices. It derived from the Fick’s second law under simplifying assumptions: uniform drug distribution, negligible matrix swelling/erosion during release, and an initial drug loading well above the drug’s solubility. In this setting, the cumulative amount released, *M_t_*, increases proportionally to the square root of time:

*M_t_* = *k_H_* × *t*^1/2^ (1)

where *k_H_* is the Higuchi constant. Because of its simplicity and clear diffusion basis, this relationship is especially appropriate for topical and transdermal systems, as well as rigid or non-swelling polymer matrices in which diffusion is the main driver.

When diffusion alone does not explain the experimental profile, researchers often turn to the Korsmeyer–Peppas equation [30] as a more adaptable empirical description. It expresses the fraction released at time “t” via a power law:

*M_t_*/*M_∞_* = *k* × *t^n^* (2)

Here, *M_∞_* is the amount released at equilibrium, *k* is a kinetic constant, and *n* is the release exponent. The exponent *n* is commonly interpreted mechanistically: *n* ≤ 0.5 is typically linked to Fickian diffusion (Higuchi-like behavior), 0.5 < *n* < 1 is often associated with anomalous transport arising from combined diffusion and polymer relaxation, and *n* ≥ 1 is often associated with release governed mainly by erosion or relaxation processes.

This model is widely applied to hydrogels, degradable polymers, and particulate carriers, where the kinetics are more complex than those of pure diffusion.

For data sets displaying a lag phase followed by a non-linear rise, the Makoid–Banakar model [31] can be a useful option combining the exponential behavior with a power-law term:

(3) $$M_{t}=M_{\infty }\times \left(1-e^{-kt^{n}}\right)$$

In this expression, *M_∞_* represents the asymptotic released amount, *k* controls the overall rate scale, and *n* adjusts the curvature. The model has proven practical for microspheres, hydrogels, and other polymeric systems where diffusion is coupled with matrix relaxation, erosion, or related structural changes.

Among these commonly used equations, the Weibull model [32] is especially valued for its flexibility and the intuitive meaning of its fitted parameters in polymeric or composite delivery matrices. In a general form it is written as

(4) $$y=A\times \left(1-e^{-{\left(k\times \left(x-x_{c}\right)\right)}^{d}}\right)$$

where *y* is the cumulative released amount (or fraction) at time *x*; *A* is the asymptotic maximum release; *k* is a time-scaling parameter associated with the apparent release rate; *x_c_* denotes a lag time; and *d* is a shape factor controlling the curvature of the profile. Fitting release data with this function yields a compact parameter set with clear interpretation. The following observations can be made: (1) The parameter *A* captures the maximum fraction that can effectively become available, which is important when release is incomplete. (2) The constant *k* reflects how rapidly the system approaches *A* and can be influenced by polymer morphology, crosslink density, and drug–matrix interactions. (3) A non-zero *x_c_* indicates an initial period in which hydration, swelling, or matrix rearrangement precedes substantial release. (4) Finally, the exponent *d* provides mechanistic clues: *d* < 1 gives a concave curve with a slower onset and more diffusion-like behavior, *d* ≈ 1 approaches an exponential form, and *d* > 1 produces a sigmoidal profile often linked to significant matrix relaxation, erosion, or degradation effects.

Release experiments were conducted in distilled water at 25 °C, with 100 mg of CLZ-loaded material dispersed in 25 mL of release medium (n = 3). Aliquots were collected at predetermined time points over a total experimental period of 180 min and analyzed by UV–Vis spectrophotometry (Thermo Scientific, Waltham, MA, USA) at 280 nm. The cumulative amount of CLZ released was determined as a function of time and subsequently evaluated using the kinetic models described above. Distilled water at 25 °C was selected as a controlled model medium for characterizing the release profile and for kinetic modeling, rather than as a simulation of physiological conditions. Sink conditions were not independently verified; therefore, the resulting profile is interpreted as an in vitro characterization of CLZ release under the specified experimental conditions.

#### 2.4.4. Antioxidant Activity

Lignin and its derivatives can be used to create materials with ultraviolet absorption, preventing harmful ultraviolet radiation, biodegradability, antibacterial, antioxidant, electron transfer, and metal adsorption properties [33].

The DPPH (2,2-diphenyl-1-picrylhydrozyl) (Sigma Aldrich; Dorset, UK) radical was employed to measure the antioxidant activity of the produced complex based on the radical scavenging property of the LIG [34]. The DPPH assay was carried out in accordance with the procedure described by Jan et al. [35]. The violet DPPH radical, stable in dimethyl sulfoxide/water solution, is transformed after its reduction in the presence of complex LCD-CLZ to a pale-yellow product, whose absorbance is measured at 517 nm. The results expressed scavenging ability (%). Firstly, 100 mL of dimethyl sulfoxide (Sigma-Aldrich; Dorset, UK)/water (10:90, *v*/*v*) was used to dissolve 2.5 mg of DPPH. LIG was dissolved in a dimethyl sulfoxide/water (10:90, *v*/*v*) solution with a concentration of 20 mg/mL. As for the effect of the complex LCD-CLZ on the antioxidant properties, a mixture was also dissolved in a dimethyl sulfoxide/water (10:90, *v*/*v*) solution with a total concentration of 75 mg/mL. Then, 50 μL of each mixture was mixed with 150 μL of the DPPH solution and made up to 3 mL with dimethyl sulfoxide/water (10:90, *v*/*v*) solution. After 60 min of dark incubation, the mixture was tested for absorbance at 517 nm in triplicate using a UV–Vis spectrophotometer (Thermo Fisher, San Jose, CA, USA). A control sample of DPPH at 0.025 mg/mL was also evaluated. The radical scavenging activity (DPPH inhibition) was calculated as Equation (5) [34].

DPPH inhibition = (Ac − As/Ac) × 100(5)

where A_c_ and A_s_ were the absorption intensities for the control and the sample probes. A_(c)_ is the absorbance of the DPPH control and A_(s)_ is the absorbance measured in the presence of the tested sample, where A_(c)_ is the absorbance of the DPPH control and A_(s)_ is the absorbance measured in the presence of the tested sample.

#### 2.4.5. Antimicrobial Activity

The antimicrobial activity of the synthesized LCD–CLZ material was evaluated against four representative microorganisms: the Gram-positive bacterium *Staphylococcus aureus* (ATCC 25923), the Gram-negative bacteria *Escherichia coli* (ATCC 25922) and *Pseudomonas aeruginosa* (ATCC 27853), and the yeast *Candida albicans* (ATCC 10311).

The compounds’ antimicrobial activity was qualitatively evaluated using the agar diffusion method, following the guidelines for antimicrobial susceptibility testing established by the Clinical and Laboratory Standards Institute (CLSI) [36]. We used the cylinder diffusion test, and the antimicrobial activity was estimated by measuring the diameter of the inhibition zone around the tested tablets. Microbial suspensions were prepared in sterile peptone water from 24-h cultures and standardized to a turbidity of 0.5 McFarland (approximately 1 × 10^81^\times 10^81^ × 10^8^ CFU/mL). These suspensions were used to inoculate 90 mm Mueller–Hinton agar plates.

The tested compounds were dissolved in dimethyl sulfoxide (DMSO) to achieve a final concentration of 600 μg/mL. Wells of 6 mm diameter were aseptically bored into the agar, and 0.1 mL of the compound solutions were carefully introduced into each well using metal cylinders placed on the agar surface. The plates were incubated at 37 °C for 24 h, allowing the compounds to diffuse into the agar and interact with the inoculated microorganisms.

Commercial fluconazole and levofloxacin susceptibility-testing disks were included as reference positive controls. Because the LCD–CLZ material and the reference antimicrobial agents were applied using different diffusion formats and at different amounts, their inhibition-zone diameters were not considered directly comparable quantitative measures of antimicrobial potency. After incubation at 37 °C for 24 h, the diameters of the inhibition zones around the wells were measured and reported as mean ± standard deviation (SD).

A solvent-only DMSO negative control was not included in the original experimental design. Therefore, a potential contribution of the solvent to the observed inhibition zones cannot be entirely ruled out, which represents a limitation of the antimicrobial assay solvent-only DMSO negative control was not included in the original experimental design. Therefore, a potential contribution of the solvent to the observed inhibition zones cannot be completely excluded, and this represents a limitation of the antimicrobial assay.

## 3. Results

### 3.1. Loading of Chlorzoxazone into LCD Matrix

**Figure 2:**
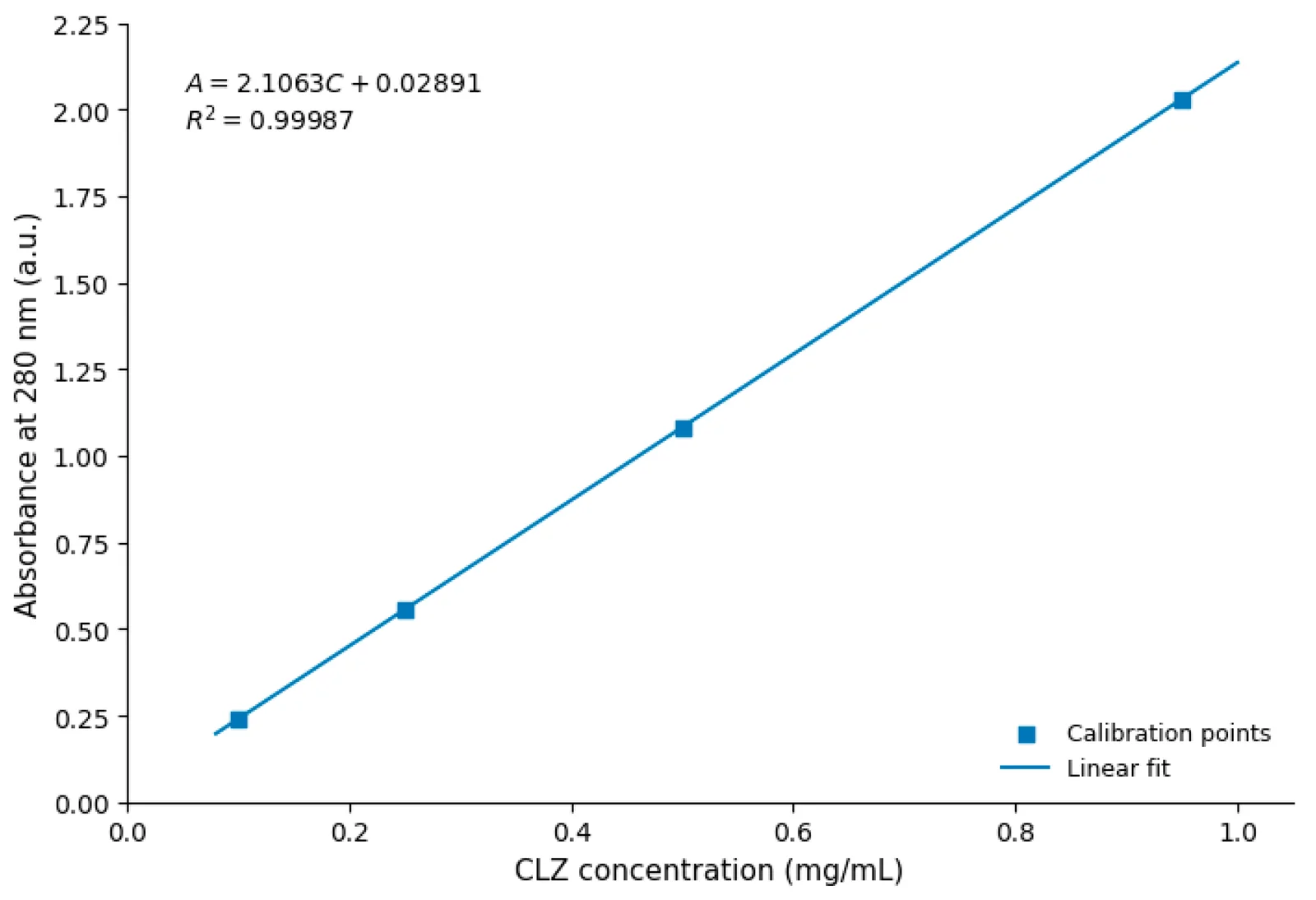
Calibration curve. Variation in the absorbance of CLZ drug loaded into LCD complex.

Based on the CLZ calibration curve (Figure 2), the amount of drug retained in the LCD matrix after the loading procedure was 41.8 mg CLZ per gram of LCD. Since 200 mg of CLZ was initially used per 1 g of LCD, the corresponding loading efficiency was 20.9%, calculated as (41.8/200) × 100. This value represents the fraction of the initially added CLZ retained by the LCD material and should be distinguished from the cumulative release fraction determined in the subsequent release experiment.

### 3.2. Fourier-Transform Infrared Spectroscopy (FTIR)

**Figure 3:**
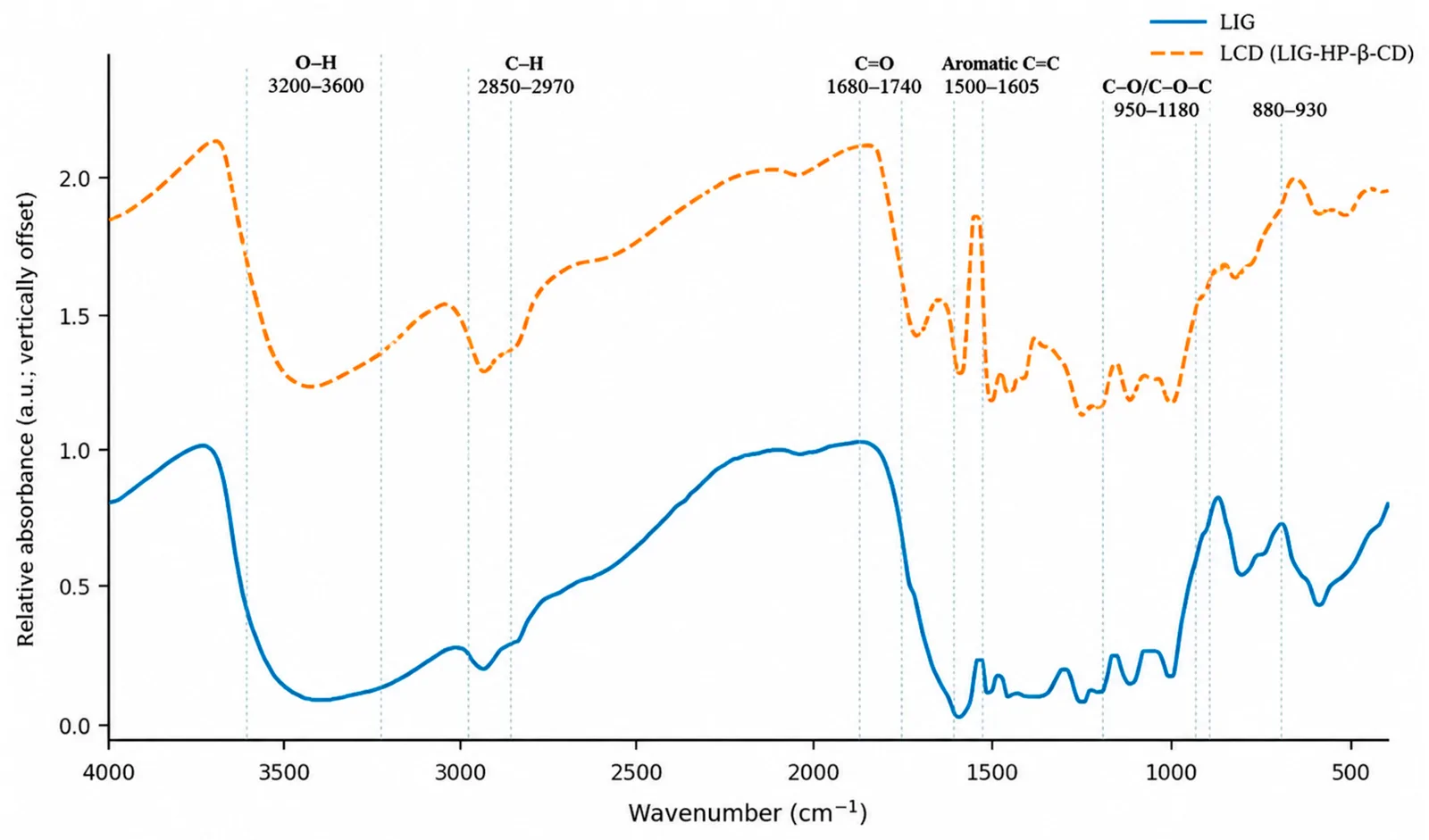
TR-FTIR spectra of native lignin (LIG) and the LIG–HP-β-CD material (LCD). Spectra are presented as relative absorbance and vertically offset for clarity. The spectral regions used for the semi-quantitative band-area analysis are indicated.

The ATR-FTIR spectra of native lignin (LIG) and the modified LIG–HP-β-CD material (LCD) are presented in Figure 3. The spectrum of native LIG displays the characteristic aromatic framework, with prominent bands in the 1500–1605 cm^−1^ region, together with a broad O–H stretching envelope at 3200–3600 cm^−1^ and contributions in the fingerprint region below 1300 cm^−1^ associated predominantly with C–O and C–O–C vibrations of phenolic, aliphatic, and ether-containing structures.

Comparison of the spectra of native LIG and LCD shows changes in the relative contributions of several oxygen-containing functional-group regions, while the characteristic aromatic lignin bands remain detectable. To describe these spectral changes semi-quantitatively, integrated band-area ratios were calculated relative to the 1500–1605 cm^−1^ aromatic region and are summarized in Table 1. These ratios are used as comparative spectral indices rather than as absolute quantitative measurements of functional-group concentrations or grafting efficiency.

The C–O/C–O–C-to-aromatic ratio increased from 1.42 in LIG to 2.98 in LCD, while the O–H-to-aromatic ratio increased from 3.54 to 4.82. The aliphatic C–H-to-aromatic ratio increased from 0.78 to 1.31. These changes are consistent with an increased relative contribution of hydroxyl-, ether-, and aliphatic-rich structures in the modified material, as expected in the presence of HP-β-CD-derived functionalities. However, because HP-β-CD itself is rich in hydroxyl and ether groups and its incorporation decreases the relative contribution of lignin to the overall spectrum, these normalized ratios cannot, on their own, distinguish covalent grafting from physical association.

The carbonyl-to-aromatic ratio also increased from 0.27 to 0.49. This observation indicates a change in the relative contribution of carbonyl-containing environments after modification; however, the present FTIR data do not permit assignment of this increase to a specific chemical mechanism. In particular, oxidative transformation of lignin cannot be established without complementary analytical evidence.

An increase was also observed in the 880–930 cm^−1^-to-aromatic ratio, from 0.06 to 0.21. Because this spectral region may contain overlapping contributions from several vibrations in the chemically heterogeneous lignin/cyclodextrin system, this change cannot be assigned specifically to residual epoxide groups or epoxide-ring opening. Accordingly, the 880–930 cm^−1^ region was not used as evidence of covalent coupling and is interpreted only as indicating a relative modification of the fingerprint region of the LCD material.

To facilitate semi-quantitative comparison of the two spectra, integrated band-area ratios were calculated relative to the 1500–1605 cm^−1^ aromatic lignin region. The selected spectral windows correspond to commonly assigned oxygen-containing and aliphatic vibrational regions in lignin- and carbohydrate-containing materials [37,38]. These ratios were used as comparative descriptors within the present dataset and should not be regarded as standardized or independently validated quantitative indices of grafting or functional-group concentration.

The spectrum of native LIG showed the characteristic broad O–H stretching band in the 3200–3600 cm^−1^ region, aliphatic C–H stretching contributions at approximately 2850–2970 cm^−1^, and the characteristic aromatic lignin bands within the 1500–1605 cm^−1^ region. In the LCD spectrum, the broad O–H region and the C–O/C–O–C fingerprint region at approximately 950–1180 cm^−1^ showed increased relative contributions, while the characteristic lignin aromatic features remained detectable. Changes were also observed in the carbonyl region at 1680–1740 cm^−1^ and in the 880–930 cm^−1^ fingerprint region. Because of extensive band overlap in the chemically heterogeneous lignin/HP-β-CD material, these spectral changes were interpreted in terms of broader regions rather than as uniquely assigned individual peaks. For a semi-quantitative comparison of the observed spectral changes, integrated FTIR band-area ratios were calculated relative to the 1500–1605 cm^−1^ aromatic lignin region, and the resulting indices are summarized in Table 1.

The reported FTIR area ratios are semi-quantitative indices intended for comparative interpretation of spectral changes and should not be considered absolute quantitative measurements of functional-group concentrations.

The FTIR area ratios reported in Table 1 should be regarded as semi-quantitative indices for comparative interpretation of spectral changes rather than as absolute quantitative measurements of functional-group concentrations. ATR-FTIR band intensities and integrated areas can be influenced by sample heterogeneity, contact with the ATR crystal, and effective penetration depth. Replicate spectra suitable for statistical estimation of uncertainty in the integrated band-area ratios were not available; consequently, these indices are reported without standard deviations and are interpreted conservatively.

It should be emphasized that the FTIR changes observed in the present study are consistent with ECH-mediated modification of lignin and the incorporation of HP-β-CD-derived functionalities, but FTIR alone cannot provide quantitative evidence of the extent of covalent grafting or rule out contributions from strongly associated components. Definitive determination of the degree of grafting and crosslinking architecture would require complementary analyses, such as solid-state NMR, elemental analysis, and/or quantitative functional-group determination, together with appropriate intermediate and physical-mixture controls. In particular, parallel characterization of HP-β-CD, the LIG–ECH intermediate, and a physical LIG/HP-β-CD mixture would be required to distinguish more rigorously between covalent modification and physical association.

### 3.3. Thermogravimetric Analysis

**Figure 4:**
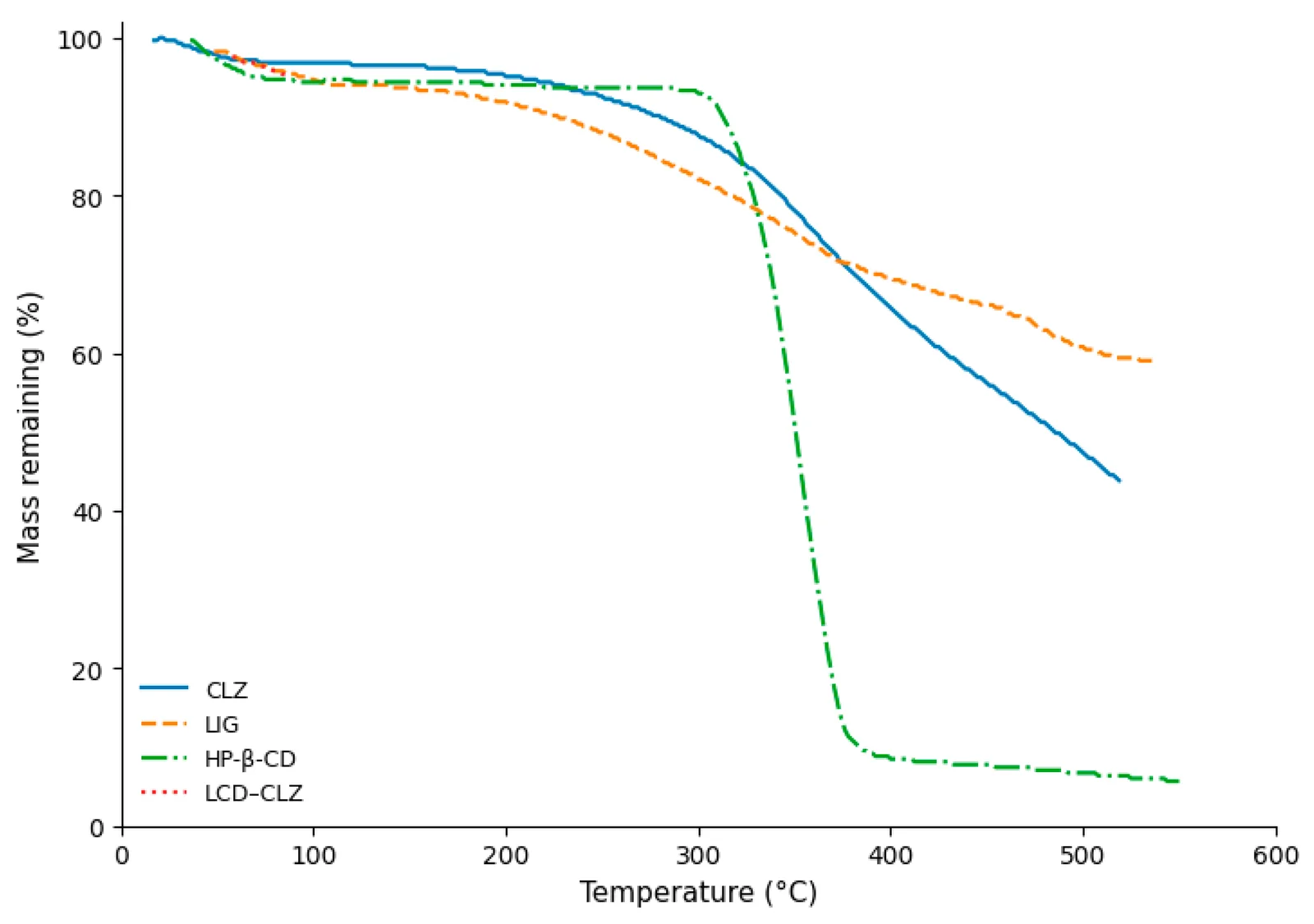
TG curves of the of native LIG, LIG–CD-CLZ conjugate (LCD), CLZ and HP-β-CD.

**Figure 5:**
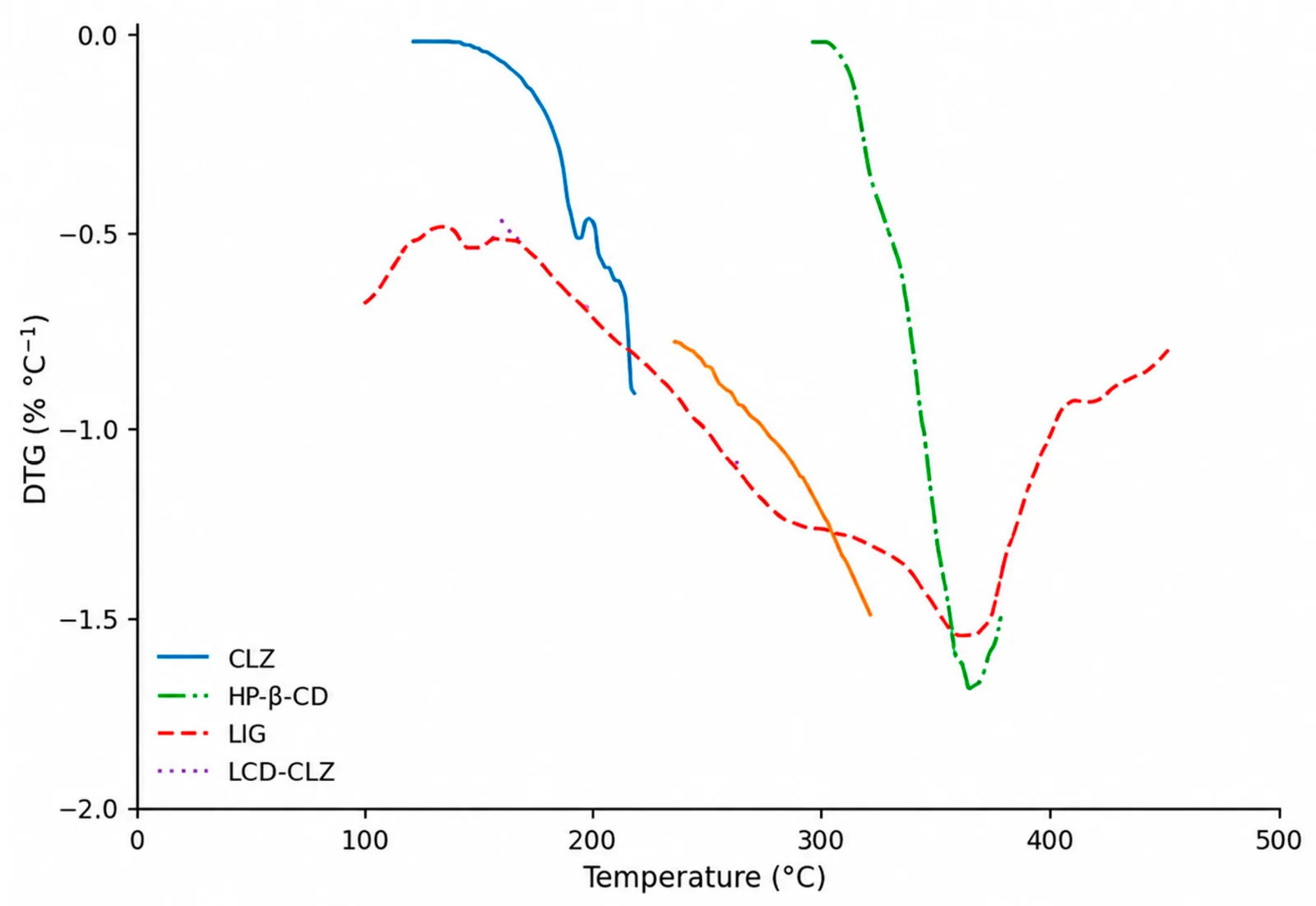
DTG curves of CLZ, HP-β-CD, native lignin (LIG), and the CLZ-loaded lignin/HP-β-CD material (LCD–CLZ).

The thermogravimetric (TG) and derivative thermogravimetric (DTG) curves are presented in Figure 4 and Figure 5, respectively.

Thermogravimetric curves (TG) and their derivative (DTG) were used to investigate the thermal behavior and possible interactions among CLZ, β-cyclodextrin (HP-β-CD), and alkaline lignin (LIG ALKAL) in their ternary system.

The TG curve of CLZ reveals high thermal stability up to approximately 200 °C, followed by a sharp mass loss in a single stage within the 200–260 °C range, with no significant residue at higher temperatures. This behavior is characteristic of a low-molecular-weight, pure organic compound that undergoes rapid decomposition.

In contrast, HP-β-CD exhibits an initial mass loss below 100–120 °C, attributed to the removal of physically adsorbed water and water bound within its cavity, followed by the main degradation stage in the 300–380 °C range, corresponding to the decomposition of the cyclodextrin structure. This two-step thermal behavior is typical of cyclodextrins and has been frequently reported in the literature [39,40,41].

Alkaline lignin shows a significantly different thermal profile, characterized by a gradual and extended mass loss over a wide temperature range (150–500 °C), reflecting its amorphous and heterogeneous structure. The high residue (~60 wt%) observed at elevated temperatures is associated with the formation of residual carbon (char), specific to the aromatic structure of lignin. This behavior is well documented and attributed to the progressive degradation of various structural fragments in lignin [42,43,44,45].

The CLZ-loaded LCD material exhibits a modified thermal profile compared with the individual components. The sharp mass-loss event characteristic of pure CLZ is not clearly resolved in the loaded material, whereas the overall mass loss occurs over a broader temperature range and is dominated by the thermal decomposition of the lignin/cyclodextrin matrix. Because CLZ represents only a relatively small fraction of the total material, the absence of a separately resolved CLZ-related event cannot, by itself, be interpreted as evidence of cyclodextrin cavity inclusion or complete molecular dispersion.

The DTG curves further illustrate this difference in thermal behavior. Pure CLZ exhibits a distinct degradation maximum at approximately 240–250 °C, whereas a separate maximum attributable to CLZ is not clearly resolved in the CLZ-loaded LCD material. This observation indicates that the thermal response of CLZ is altered or obscured in the presence of the polymeric matrix. However, given the relatively low CLZ content and the broad decomposition profile of the LCD matrix, overlap or masking of the CLZ thermal event cannot be excluded.

The broad degradation region observed at higher temperatures in the loaded material overlaps with the principal decomposition domains of HP-β-CD and lignin. Although interactions between CLZ and the surrounding matrix may contribute to the altered thermal profile, the present TG–DTG data do not permit the individual contribution of CLZ to be separated from matrix decomposition. Therefore, a specific increase in the thermal stability of CLZ or a defined host–guest inclusion mechanism cannot be established from these data alone.

Overall, the TG–DTG results demonstrate that the thermal behavior of the CLZ-loaded LCD material differs from that of the individual components and that a distinct CLZ degradation event is not resolved in the loaded system. These findings are consistent with interactions between CLZ and the lignin/cyclodextrin matrix, but they do not independently demonstrate inclusion of CLZ within the HP-β-CD cavity or distinguish molecular dispersion from a physical mixture. Comparison with unloaded LCD and an LCD/CLZ physical mixture of equivalent composition, together with complementary DSC and powder X-ray diffraction analyses, would be required for a more definitive structural interpretation.

### 3.4. Chlorzoxazone Release from LCD Inclusion Complex

Both the Weibull and Makoid–Banakar models provided a close description of the experimental CLZ release profile. The Makoid–Banakar model yielded a marginally lower reduced χ^2^, whereas the Weibull model provided a slightly higher coefficient of determination (R^2^ = 0.99394 versus 0.99319). Given the small differences between these goodness-of-fit measures, the present data do not support an unequivocal statistical preference for one model over the other. The Weibull model was retained as a convenient empirical descriptor of the release profile because its fitted parameters provide a compact representation of the asymptotic release level and curve shape (Table 2).

**Figure 6:**
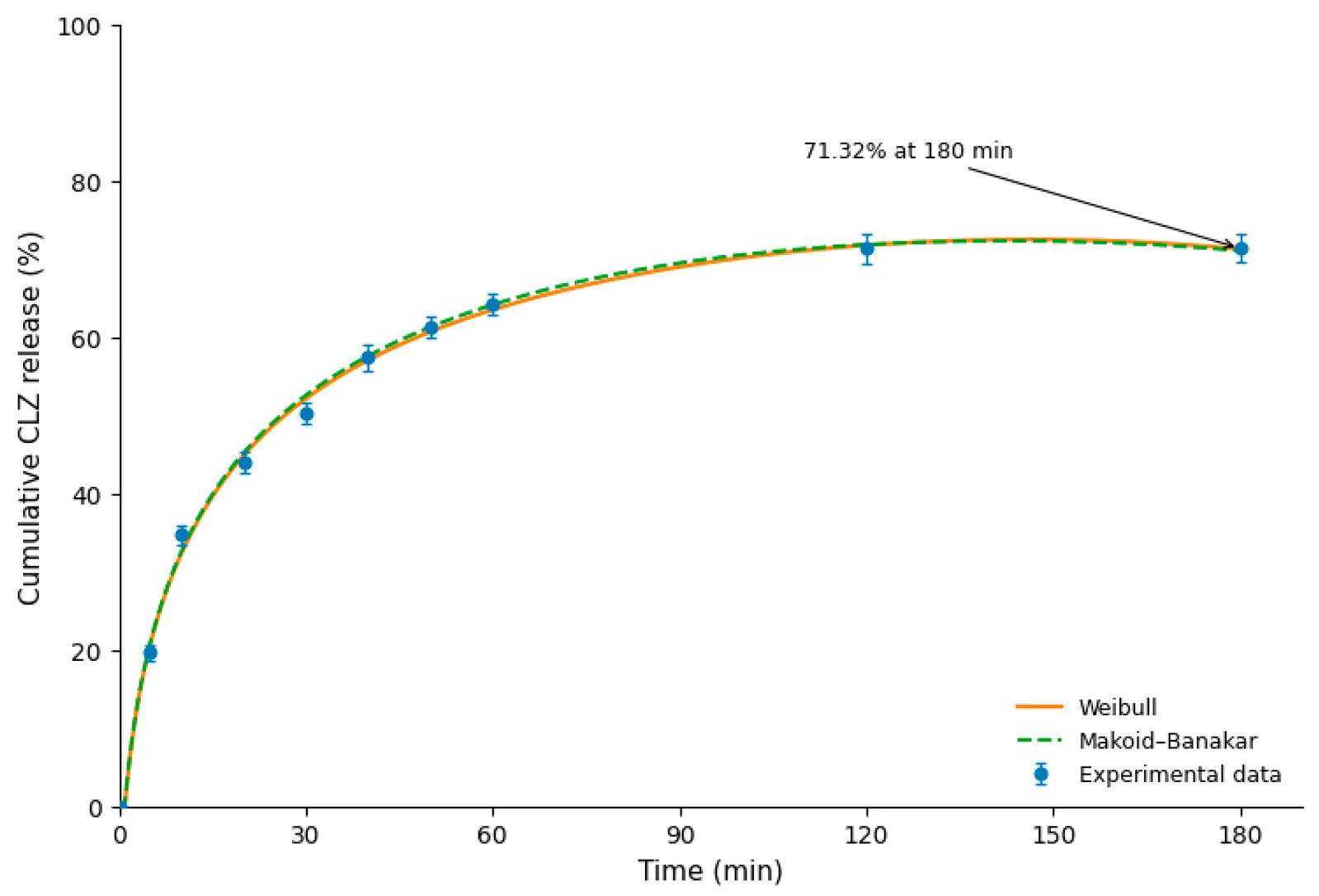
Cumulative CLZ release from the LCD–CLZ material expressed as percentage of the experimentally determined loaded drug, together with the Weibull and Makoid–Banakar fitted profiles. Data are presented as mean ± SD (n = 3).

The reduced χ^2^ values of the Weibull and Makoid–Banakar models were very similar, indicating comparable goodness of fit. Although the Makoid–Banakar model yielded a marginally lower reduced χ^2^, the Weibull model showed a slightly higher R^2^. These small differences should not be interpreted as demonstrating statistical superiority of either model. Accordingly, model selection was not based on a single goodness-of-fit parameter. The Weibull model was retained primarily as a convenient empirical representation of the experimental release profile, while the Makoid–Banakar model was considered to provide an essentially comparable fit. Reduced chi-square represents the chi-square statistic normalized by the degrees of freedom and therefore expresses the average weighted squared deviation between the experimental points and the fitted curve (Figure 6). It is most discriminating when the point-by-point experimental uncertainties used for weighting are accurately known; when uncertainty estimates are approximate or not strictly uniform across the time domain, small differences in *red-χ*^2^ can reflect the weighting scheme as much as genuine differences in model adequacy. For this reason, model selection was based on a combined assessment of statistical performance and interpretability across the full release profile. The Weibull equation provided the highest coefficient of determination, indicating the most consistent description of variance over the entire dataset, and, importantly, returned parameters with direct physical meaning for polymeric and composite delivery matrices. The asymptotic parameter *A* closely matched the experimentally observed plateau, confirming that the model captures the incomplete but well-defined maximum attainable release from the LIG-HP-β-CD carrier, while a negligible lag time *x_c_* supported the absence of a pronounced induction period prior to release onset. The shape factor *d* was below unity, consistent with the concave-down release curve observed experimentally, where a rapid initial liberation is followed by a progressively slower approach to equilibrium. This behavior is compatible with an early phase dominated by release of a readily accessible or weakly retained drug, and a later phase governed by decreasing concentration gradients and longer effective diffusion pathways within the matrix and inclusion domains. In this sense, the Weibull function not only reproduces the kinetics with excellent goodness-of-fit, but also provides a compact and mechanistically readable parametrization of the time-dependent release process, making it particularly suitable for reporting and for comparing formulation-dependent changes in release performance.

As can be seen from Figure 6, CLZ release starts immediately and rises steeply in the early stage (a “burst effect high-speed release”), reaching roughly 8–15 mg/g within the first 10–15 min, then continuing to increase but with a clearly reduced slope extended-release (transition to a “slow phase”). By around ~40–60 min, the system approaches 24–27 mg/g, after which the curve gradually flattens, reaching an apparent plateau near 30 mg/g by 120–180 min.

This two-stage pattern (fast initial liberation of more accessible/loosely retained CLZ followed by slower approach to an asymptote as diffusion paths lengthen and the concentration gradient weakens) is captured slightly more faithfully by Weibull than by Makoid–Banakar, as reflected by the better fit statistics and by the physically meaningful Weibull parameters (especially *A*, *x_c_*, and *d*).

After 180 min, the cumulative amount of CLZ released was 29.81 mg/g, corresponding to 71.32% of the experimentally determined loaded CLZ amount (41.8 mg/g). Thus, complete release was not achieved within the 180 min experimental period.

### 3.5. Antioxidant Activity

The antioxidant response of the investigated materials was evaluated using the DPPH radical-scavenging assay. Lignin-derived phenolic functionalities are known to contribute to radical-scavenging activity through electron- or proton-transfer mechanisms [34].

Under the experimental conditions used, LIG tested at 20 mg/mL showed a DPPH inhibition of 41.82 ± 1.07%, whereas LCD–CLZ tested at 75 mg/mL showed an inhibition of 50.32 ± 1.05%. These results demonstrate measurable radical-scavenging activity for both materials under their respective experimental conditions. However, because LIG and LCD–CLZ were evaluated at different sample concentrations, the numerical difference between these values cannot be interpreted as evidence of enhanced intrinsic antioxidant activity following HP-β-CD modification or CLZ loading.

The radical-scavenging response observed for the lignin-containing materials is consistent with the presence of antioxidant functionalities, particularly lignin-derived phenolic groups. Nevertheless, the present single-concentration measurements do not allow quantitative comparison of antioxidant potency or determination of the specific contribution of CLZ loading. Equivalent-concentration measurements and concentration–response analysis would be required to establish such differences.

### 3.6. Antimicrobial Activity

**Figure 7:**
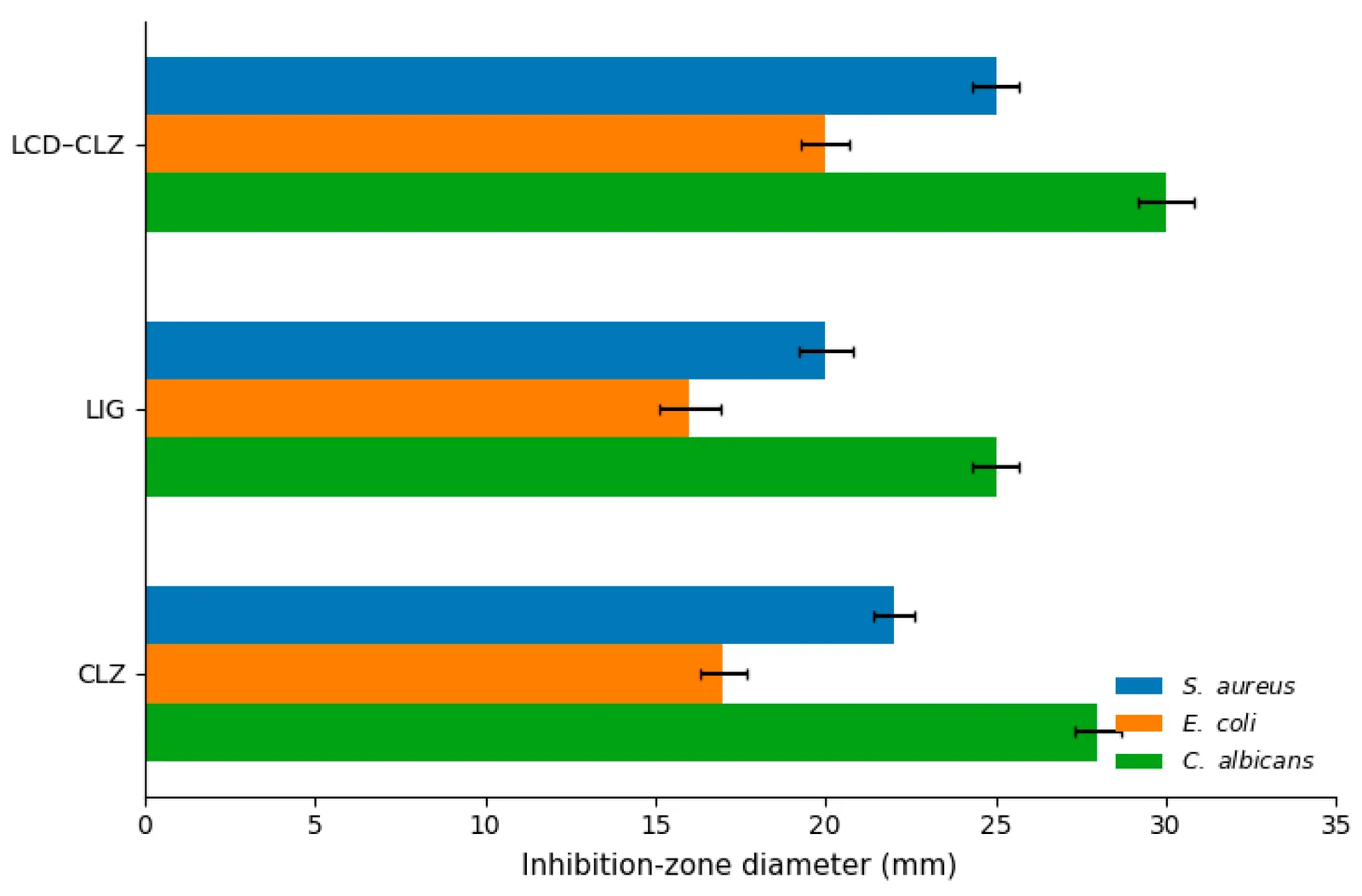
In vitro antimicrobial activity of LCD–CLZ, LIG, and CLZ against the tested microorganisms, expressed as inhibition-zone diameter (mm). Data are presented as mean ± SD.

The antimicrobial activity of the LCD–CLZ material was evaluated against *Staphylococcus aureus*, *Escherichia coli*, *Pseudomonas aeruginosa*, and *Candida albicans* using the agar-diffusion assay described in Section 2.4.5. The results of the antimicrobial susceptibility tests are presented in Figure 7.

Under the experimental conditions used, the LCD–CLZ material at 600 μg/mL produced measurable inhibition zones against *S. aureus*, *E. coli*, and *C. albicans*, whereas no effective inhibition was observed against *P. aeruginosa*. Among the susceptible microorganisms tested, the largest inhibition response was observed for *C. albicans*. These results indicate microorganism-dependent in vitro antimicrobial activity of the LCD–CLZ material under the conditions of the agar-diffusion assay.

The antimicrobial response may be partly associated with lignin-derived phenolic functionalities, which have previously been associated with disruption of microbial membrane integrity and other antimicrobial effects [46,47]. However, the present assay does not establish the specific mechanism responsible for the observed inhibition, and the contribution of the individual components of the LCD–CLZ material cannot be determined from these results alone.

Commercial fluconazole and levofloxacin disks were included as reference positive controls for the assay. Because the LCD–CLZ material and the reference antimicrobial agents were applied using different diffusion formats and at different amounts, their inhibition-zone diameters were not considered directly comparable quantitative measures of antimicrobial potency. Accordingly, the antimicrobial findings should be interpreted as qualitative to semi-quantitative evidence of in vitro activity rather than as a direct comparison with standard antimicrobial drugs.

In addition, because a solvent-only DMSO negative control was not included in the original experimental design, a potential contribution of the solvent to the observed inhibition zones cannot be completely excluded. This represents a limitation of the antimicrobial assay and should be addressed in subsequent studies using appropriate solvent controls and quantitative antimicrobial methods. Because an unloaded LCD control was not evaluated in parallel, the present results do not allow the specific contribution of CLZ loading to the antimicrobial response to be determined.

Overall, the LCD–CLZ material exhibited microorganism-dependent inhibitory activity under the experimental conditions used. Further studies employing appropriate negative controls and quantitative methods, including minimum inhibitory concentration (MIC) and minimum bactericidal/fungicidal concentration measurements, would be required to establish antimicrobial potency and better characterize the contribution of the individual matrix components.

The present study represents a preliminary characterization of the LCD–CLZ system and does not establish its suitability as a pharmaceutical dosage form. In particular, residual epichlorohydrin was not quantitatively determined, and cytocompatibility, biocompatibility, and in vivo safety were not evaluated. These aspects, together with formulation-specific studies including dosage-form preparation, mechanical properties, disintegration, dissolution under physiologically relevant conditions, and stability, should be addressed before pharmaceutical application can be considered.

## 4. Discussions

### 4.1. CLZ Loading and Drug–Matrix Association

The present study investigated an ECH-mediated lignin/HP-β-CD material as a matrix for the loading and release of poorly water-soluble CLZ. The experimentally determined CLZ loading was 41.8 mg/g, corresponding to a loading efficiency of 20.9% relative to the initially added drug. This finding demonstrates retention of CLZ by the LCD matrix under the experimental loading conditions; however, it should not be interpreted as a measure of equilibrium binding capacity or as direct evidence of host–guest inclusion.

Importantly, cyclodextrin complexation of CLZ itself has previously been demonstrated. Tang et al. investigated CLZ complexes with both β-CD and HP-β-CD and reported improved dissolution characteristics, particularly for HP-β-CD. Their complexes were characterized using a combination of FTIR, PXRD, ^1H NMR, DSC, and SEM [48]. This previous work is particularly relevant because it demonstrates that CLZ is capable of interacting with HP-β-CD, but it also highlights an important distinction from the present study: here, HP-β-CD is incorporated into a lignin-based matrix rather than investigated as an isolated CLZ–cyclodextrin system.

Lignin-based materials are increasingly investigated as drug carriers because their aromatic structure, functional-group diversity, and tunable physicochemical characteristics permit association with a variety of bioactive molecules [49,50]. The present loading result therefore extends established CLZ–cyclodextrin chemistry into a more complex lignin/HP-β-CD matrix. Further optimization of matrix composition, HP-β-CD content, drug-to-matrix ratio, and loading conditions would be required to determine whether loading efficiency can be improved.

### 4.2. CLZ Release Behavior and Kinetic Modelling

After 180 min, cumulative CLZ release reached 29.81 mg/g, corresponding to 71.32% of the experimentally determined loaded drug. The release profile showed a relatively rapid initial phase followed by a progressively slower approach to an apparent plateau. This indicates that a fraction of the loaded CLZ remained associated with the matrix during the investigated period.

Cyclodextrin-based systems can modify the apparent dissolution and release behavior of poorly water-soluble drugs through reversible drug–cyclodextrin association, while incorporation of cyclodextrins into polymeric structures introduces additional matrix-dependent constraints on drug transport [51]. In the specific case of CLZ, Tang et al. demonstrated enhanced dissolution following complexation with β-CD and HP-β-CD [48]. The present system differs because release occurs from a lignin/HP-β-CD matrix, where both cyclodextrin association and interactions with the polymeric environment may influence the observed profile.

Both Weibull and Makoid–Banakar models provided closely comparable descriptions of the experimental release data. Weibull showed a slightly higher R^2^ (0.99394 versus 0.99319), whereas Makoid–Banakar yielded a marginally lower reduced χ^2^. These differences are too small to establish unequivocal statistical superiority of either model. Accordingly, Weibull was retained as a convenient empirical descriptor rather than as evidence of a specific molecular release mechanism.

The fitted kinetic parameters should likewise be interpreted cautiously. Empirical release models describe the shape of the release curve but do not independently distinguish diffusion, matrix relaxation, swelling, erosion, or combinations of these processes. Furthermore, because release was evaluated in distilled water at 25 °C and sink conditions were not independently verified, the present findings should be regarded as characterization under controlled experimental conditions rather than as a prediction of physiological dissolution behavior.

From a pharmaceutical perspective, the observed release profile should not be considered a prolonged-release profile. Although complete CLZ release was not achieved within 180 min, approximately 71.32% of the loaded drug was released during this period, with a pronounced initial release phase. Such behavior may be useful when relatively rapid drug availability is desired, but it would be suboptimal for applications requiring sustained delivery over extended periods. The initial burst may reflect release of drug located at or near the matrix surface or more weakly associated with accessible regions of the carrier; however, the present experiments do not allow these contributions to be distinguished mechanistically.

Future optimization should therefore focus on reducing the initial burst and extending the release period. Potential strategies include increasing matrix crosslinking density, modifying the lignin/HP-β-CD ratio, increasing matrix particle size or reducing accessible surface area, optimizing the drug-loading procedure to reduce superficially associated CLZ, and incorporating the LCD–CLZ material into a secondary polymeric or hydrogel matrix. These modifications would require systematic evaluation because they may simultaneously affect drug loading, diffusion pathways, matrix hydration, and overall release kinetics.

### 4.3. FTIR Characterization

The FTIR analysis showed clear differences between native LIG and the modified LCD material. The C–O/C–O–C-to-aromatic ratio increased from 1.42 to 2.98, the O–H-to-aromatic ratio from 3.54 to 4.82, and the aliphatic C–H-to-aromatic ratio from 0.78 to 1.31. These changes are consistent with an increased relative contribution of hydroxyl-, ether-, and aliphatic-rich structures, and therefore with the presence of HP-β-CD-derived functionalities.

However, the normalized ratios cannot independently establish covalent grafting. Incorporation of an HP-β-CD-rich component necessarily changes the relative lignin contribution to the overall spectrum, particularly when lignin aromatic bands are used as the denominator. The carbonyl/aromatic increase from 0.27 to 0.49 similarly indicates a change in relative spectral composition but does not establish oxidative transformation of lignin. The 880–930 cm^−1^ region also contains overlapping contributions and cannot independently demonstrate epoxide-ring opening.

This limitation is consistent with established analytical principles for cyclodextrin systems. Characterization of true inclusion complexes generally requires complementary analytical approaches rather than reliance on a single spectroscopic technique [52,53]. Tang et al., for example, combined FTIR with PXRD, ^1H NMR, DSC, and SEM when characterizing CLZ–cyclodextrin complexes [48].

Consequently, the present FTIR findings support modification of the lignin-based material and incorporation of HP-β-CD-derived functionalities but do not independently establish the degree or architecture of covalent grafting.

### 4.4. Thermal Behavior

TG/DTG analysis showed that the thermal profile of LCD–CLZ differed from those of the individual components. Pure CLZ exhibited a distinct degradation maximum at approximately 240–250 °C, whereas a separate CLZ-related maximum was not clearly resolved in the loaded material.

Although changes or disappearance of drug-related thermal events may accompany cyclodextrin complex formation, thermal analysis alone is insufficient to establish inclusion, particularly in heterogeneous solid matrices. Reviews of cyclodextrin characterization emphasize the complementary roles of DSC, PXRD, spectroscopy, and NMR in distinguishing physical mixtures from true inclusion systems [52,53]. The CLZ–HP-β-CD study by Tang et al. similarly relied on several complementary techniques rather than thermal analysis alone [48].

In the present system, the relatively low CLZ content and broad thermal decomposition of the lignin/HP-β-CD matrix could mask a separate CLZ thermal event. The TG/DTG findings are therefore consistent with an altered thermal environment of CLZ but do not independently demonstrate molecular dispersion or inclusion within the HP-β-CD cavity. DSC and PXRD, together with unloaded LCD and an LCD/CLZ physical-mixture control, would substantially strengthen this interpretation.

### 4.5. Antioxidant Properties

Lignin is well recognized as a source of antioxidant functionality because of its phenolic structures and ability to participate in radical-scavenging reactions. These properties have contributed to increasing interest in lignin and lignin-derived materials for biomedical and drug-delivery applications [49,50].

In the present study, LIG tested at 20 mg/mL produced 41.82 ± 1.07% DPPH inhibition, whereas LCD–CLZ tested at 75 mg/mL produced 50.32 ± 1.05%. These results demonstrate measurable radical-scavenging activity under the respective experimental conditions. However, because the samples were evaluated at different concentrations, their numerical difference cannot establish enhanced intrinsic antioxidant potency following modification or CLZ loading.

The results should therefore be interpreted as preliminary evidence that antioxidant functionality is retained in the lignin-containing system. Equivalent-concentration measurements and concentration–response analysis would be required to establish whether preparation of LCD or loading with CLZ quantitatively alters antioxidant potency.

### 4.6. Antimicrobial Properties

LCD–CLZ exhibited microorganism-dependent activity under the agar-diffusion conditions. Measurable inhibition was observed against *S. aureus*, *E. coli*, and *C. albicans*, whereas no effective inhibition was observed against *P. aeruginosa*.

Antibacterial properties of lignin and lignin-derived materials have been increasingly investigated, with polyphenolic functionality and interactions with bacterial envelopes among the mechanisms proposed in the literature [54]. Lignin-based materials are consequently being explored for antimicrobial biomedical applications, as well as drug-delivery platforms [49,54].

Nevertheless, the present experiment does not permit the antimicrobial contribution of CLZ loading to be isolated because unloaded LCD was not evaluated in parallel. Moreover, agar-diffusion zone size depends on both biological activity and diffusion through the agar matrix. The absence of a solvent-only DMSO control represents an additional limitation.

Commercial fluconazole and levofloxacin disks should therefore be regarded only as reference positive controls rather than quantitatively comparable standards. Furthermore, because only standard ATCC strains were evaluated, the present findings cannot be extrapolated to multidrug-resistant clinical isolates.

Comparison of the inhibition-zone diameters also indicates that incorporation of CLZ into the LCD material did not produce a uniformly pronounced enhancement of antimicrobial activity relative to free CLZ. Although some differences were observed among the tested microorganisms, the magnitude of these differences was limited and microorganism-dependent. Therefore, the present data do not support a general synergistic or additive antimicrobial effect resulting from CLZ loading into the LCD matrix.

This observation is consistent with the fact that antimicrobial performance in cyclodextrin- and polymer-based systems depends not only on the intrinsic activity of the incorporated compound, but also on its availability and release from the carrier, as well as on diffusion of the material through the assay medium. Similar cyclodextrin-containing antimicrobial delivery systems have been investigated as strategies for modifying the physicochemical availability of incorporated antimicrobial compounds [52]. However, the present agar-diffusion experiment was not designed to establish synergy between CLZ and the LCD matrix.

### 4.7. Specific Advance of the Present System

The novelty of the present study should be distinguished from the broader concepts of cyclodextrin complexation and lignin-based drug delivery, both of which are already established. Contemporary reviews demonstrate the extensive development of cyclodextrin-based drug-delivery scaffolds and chemically modified cyclodextrin systems [51]. Likewise, lignin-derived carriers have been investigated across a growing range of biomedical applications [49,50].

Most importantly, CLZ complexation with β-CD and HP-β-CD has already been reported [48]. Therefore, the advance of the present work is not the first demonstration of CLZ–HP-β-CD interaction. Rather, it lies in extending CLZ delivery to an ECH-mediated lignin/HP-β-CD matrix and evaluating, within the same system, CLZ loading, release kinetics, FTIR and thermal characteristics, and preliminary antioxidant and antimicrobial behavior.

This distinction provides a more precise definition of the study’s contribution and avoids overstating novelty. The present work should consequently be viewed as an initial characterization of a multifunctional lignin/HP-β-CD platform for CLZ rather than as demonstration of an entirely new drug-delivery concept.

### 4.8. Limitations and Future Directions

Several limitations should be considered. The degree of HP-β-CD grafting, crosslinking architecture, and residual ECH content were not quantitatively determined. FTIR provided semi-quantitative evidence of compositional modification but could not establish covalent architecture independently. As emphasized in the analytical literature on cyclodextrin complexes, robust structural characterization requires complementary analytical methods [52,53].

TG/DTG alone similarly cannot establish CLZ inclusion or molecular dispersion. DSC, PXRD, solid-state NMR, unloaded LCD, and appropriate physical-mixture controls should therefore be incorporated into subsequent studies. This is particularly relevant because previous CLZ–HP-β-CD work employed multiple complementary methods to establish inclusion [48].

Equilibrium CLZ solubility was not directly measured, sink conditions were not independently verified, and release was evaluated in distilled water at 25 °C. Future experiments should directly assess equilibrium solubility and dissolution/release under pharmaceutically and physiologically relevant conditions.

The biological assays were exploratory. Equivalent-concentration and concentration–response antioxidant experiments are needed, while antimicrobial studies should include unloaded LCD and solvent controls together with MIC and minimum bactericidal/fungicidal concentration measurements. Finally, residual-reagent safety, cytocompatibility, biocompatibility, stability, in vivo behavior, and dosage-form performance require evaluation before pharmaceutical application can be considered.

## 5. Conclusions

An ECH-mediated lignin/HP-β-CD material was investigated as a matrix for the loading and release of poorly water-soluble chlorzoxazone. The experimentally determined CLZ loading was 41.8 mg/g of LCD, corresponding to a loading efficiency of 20.9% relative to the initially added drug. After 180 min, cumulative CLZ release reached 29.81 mg/g, representing 71.32% of the loaded CLZ. Both the Weibull and Makoid–Banakar models provided closely comparable descriptions of the experimental release profile, without unequivocal statistical superiority of either model.

FTIR analysis showed changes in the relative spectral composition of the modified lignin-based material consistent with the presence of HP-β-CD-derived functionalities. TG/DTG analysis demonstrated that the thermal profile of the CLZ-loaded LCD material differed from those of the individual components. These findings support modification of the lignin-based matrix and drug–matrix association but do not independently establish the extent of covalent HP-β-CD grafting, molecular dispersion of CLZ, or inclusion of CLZ within the HP-β-CD cavity.

The lignin-containing materials showed measurable radical-scavenging activity under the experimental conditions, although the different concentrations used for LIG and LCD–CLZ preclude direct quantitative comparison of antioxidant potency. LCD–CLZ also exhibited microorganism-dependent inhibitory activity against *Staphylococcus aureus*, *Escherichia coli*, and *Candida albicans*, whereas no effective inhibition was observed against *Pseudomonas aeruginosa*.

Future studies should focus on optimizing the lignin/HP-β-CD composition and crosslinking conditions to increase CLZ loading efficiency, reduce the initial burst release, and extend the release period. Structural validation should include appropriate intermediate and physical-mixture controls, together with complementary techniques such as DSC, PXRD, and solid-state NMR. Direct determination of equilibrium CLZ solubility, residual ECH, cytocompatibility, and biocompatibility will also be required. Finally, release studies under pharmaceutically relevant conditions and subsequent formulation into an appropriate dosage form will be necessary to determine whether the LCD–CLZ platform has practical pharmaceutical potential.

## Figures and Tables

**Table 1:** Integrated FTIR band-area indices used for comparing LIG and LIG–ECH–CD conjugate.

Index (Area Ratio of Different Spectral Regions in Respect with Aromatic Ones)	LIG	LCD
Carbonyl/aromatic (1680–1740)/(1500–1605)	0.27	0.49
Aliphatic C–H/aromatic (2850–2970)/(1500–1605)	0.77	1.30
O–H/aromatic (3200–3600)/(1500–1605)	3.53	4.81
C–O region/aromatic (950–1180)/(1500–1605)	1.42	2.97
880–930 region/aromatic (880–930)/(1500–1605)	0.06	0.20

**Table 2:** Kinetic model fitting parameters define the optimal descriptor of CLZ release from LCD inclusion complex.

**Weibull**	*A*	30.64107 ± 0.92093	**Makoid–Banakar**	*k*	4.50694 ± 0.45278
	*x_c_*	−2.92273 × 10^−20^ ± 2.30113 × 10^−7^		*a*	0.00347 ± 4.84411 × 10^−4^
	*d*	0.68517 ± 0.05257		*n*	0.48338 ± 0.03273
	*k*	0.0436 ± 0.00439		*red-χ* ^2^	0.56574
	*red-χ* ^2^	0.63631		R^2^	0.99394
	R^2^	0.99319			
**Korsmeyer–Peppas**	*k*	7.96067 ± 1.1239	**Higuchi**	*k*	2.94505 ± 0.22255
	*n*	0.27311 ± 0.03338		*red-χ* ^2^	25.50732
	*red-χ* ^2^	4.57362		R^2^	0.72693
	R^2^	0.95104			

## Data Availability

The original contributions presented in this study are included in the article. Further inquiries can be directed to the corresponding authors.

## Abbreviations

The following abbreviations are used in this manuscript:

CLZ	Chlorzoxazone
LIG	Lignin
HP-β-CD	Hydroxypropyl-β-cyclodextrin
CD	Cyclodextrin
LCD	Lignin–hydroxypropyl-β-cyclodextrin conjugate
LCD-CLZ	Chlorzoxazone-loaded lignin–hydroxypropyl-β-cyclodextrin complex
ECH	Epichlorohydrin
FTIR	Fourier-transform infrared spectroscopy
ATR	Attenuated total reflectance
TGA	Thermogravimetric analysis
TG	Thermogravimetry
DTG	Derivative thermogravimetry
DPPH	2,2-diphenyl-1-picrylhydrazyl
DMSO	Dimethyl sulfoxide
UV–Vis	Ultraviolet–visible spectroscopy
BCS	Biopharmaceutics Classification System
CLSI	Clinical and Laboratory Standards Institute
CFU	Colony-forming unit
SD	Standard deviation
